# The microRNA-34a-Induced Senescence-Associated Secretory Phenotype (SASP) Favors Vascular Smooth Muscle Cells Calcification

**DOI:** 10.3390/ijms21124454

**Published:** 2020-06-23

**Authors:** Estella Zuccolo, Ileana Badi, Francesco Scavello, Irene Gambuzza, Luigi Mancinelli, Federica Macrì, Calogero C. Tedesco, Fabrizio Veglia, Anna Rita Bonfigli, Fabiola Olivieri, Angela Raucci

**Affiliations:** 1 Unit of Experimental Cardio-Oncology and Cardiovascular Aging, Centro Cardiologico Monzino-IRCCS, 20138 Milan, Italy; Estella.Zuccolo@cardiologicomonzino.it (E.Z.); ileana.badi@cardiov.ox.ac.uk (I.B.); Francesco.Scavello@cardiologicomonzino.it (F.S.); irenegambuzza94@gmail.com (I.G.); jkducati695@gmail.com (L.M.); Federica.Macri@cardiologicomonzino.it (F.M.); 2 Unit of Biostatistics, Centro Cardiologico Monzino-IRCCS, 20138 Milan, Italy; Calogero.Tedesco@cardiologicomonzino.it (C.C.T.); Fabrizio.Veglia@cardiologicomonzino.it (F.V.); 3 Scientific Direction, IRCCS INRCA, 60124 Ancona, Italy; A.BONFIGLI@inrca.it; 4 Department of Clinical and Molecular Sciences, DISCLIMO, Università Politecnica delle Marche, 60020 Ancona, Italy; f.olivieri@univpm.it; 5 Center of Clinical Pathology and Innovative Therapy, IRCCS INRCA, 60127 Ancona, Italy

**Keywords:** inflammaging, vascular calcification, VSMCs, senescence, SASP, IL6

## Abstract

The senescence of vascular smooth muscle cells (VSMCs), characterized by the acquisition of senescence-associated secretory phenotype (SASP), is relevant for VSMCs osteoblastic differentiation and vascular calcification (VC). MicroRNA-34a (miR-34a) is a driver of such phenomena and could play a role in vascular inflammaging. Herein, we analyzed the relationship between miR-34a and the prototypical SASP component IL6 in in vitro and in vivo models. miR-34a and IL6 levels increased and positively correlated in aortas of 21 months-old male C57BL/6J mice and in human aortic smooth muscle cells (HASMCs) isolated from donors of different age and undergone senescence. Lentiviral overexpression of miR-34a in HASMCs enhanced IL6 secretion. HASMCs senescence and calcification accelerated after exposure to conditioned medium of miR-34a-overexpressing cells. Analysis of miR-34a-induced secretome revealed enhancement of several pro-inflammatory cytokines and chemokines, including IL6, pro-senescent growth factors and matrix-degrading molecules. Moreover, induction of aortas medial calcification and concomitant IL6 expression, with an overdose of vitamin D, was reduced in male C57BL/6J *Mir34a^−/−^* mice. Finally, a positive correlation was observed between circulating miR-34a and IL6 in healthy subjects of 20-90 years. Hence, the vascular age-associated miR-34a promotes VSMCs SASP activation and contributes to arterial inflammation and dysfunctions such as VC.

## 1. Introduction

The deterioration of arterial anatomy and physiology that occurs during chronological aging is a risk factor for cardiovascular morbidity and all-cause mortality [1]. Aged arteries are characterized by functional changes of vascular smooth muscle cells (VSMCs) from a contractile and quiescent status to a senescent phenotype distinguishable for an enlarged morphology, loss of contraction, irreversible growth arrest, increased activity of senescence-associated β-galactosidase (SA-β-gal) and enhanced expression the cyclin-dependent kinase inhibitors p21 and p16 and the tumor suppressor gene p53 [2,3,4,5]. Besides, VSMCs approaching senescence, acquire the senescence-associated secretory phenotype (SASP) that consists of the secretion of a variety of soluble molecules mostly pro-inflammatory cytokines and chemokines, growth factors and matrix-remodeling enzymes [6,7,8]. SASP factors are released in the blood circulation and act locally in a paracrine manner to spread senescence to neighboring cells [7,9]; in this way, they contribute to the development of a sterile, low-grade, chronic age-associated systemic and tissues inflammation known as “inflammaging” considered the main risk factor for the most common age related diseases, included cardiovascular diseases [10,11,12].

Senescent VSMCs express also bone-related genes, like Runt-related transcription factor 2 (Runx2), alkaline phosphatase and osteocalcin that favor their maladaptive switching to an osteoblastic phenotype and eventually, the onset of vascular calcification (VC), a cardiovascular complication characterized by hydroxyapatite crystals deposition and mineralization of the arterial wall [13,14,15]. Accordingly, during aging or in pathological conditions including chronic kidney disease (CKD), atherosclerosis or type 2 diabetes (T2D), the molecular mechanisms that promote VSMCs senescence, like prelamin A accumulation [16] or cartilage oligomeric matrix protein (COMP) [17], sirtuin 1 (SIRT1) [18] and AXL receptor tyrosine kinase (Axl) [19] downregulation, support their osteogenic transdifferentiation and VC. Notably, arterial inflammaging is a driver of VC as well since many SASP molecules such as Interleukin 6 (IL6), bone morphogenetic protein 2 (BMP2) and osteoprotegerin (OPG) are known to reinforce osteoblastic transition of nearby senescent VSMCs [16,20]. Unraveling the molecular mechanisms by which senescent VSMCs can trigger inflammaging could help to develop effective treatments for the age-related diseases including VC.

MicroRNAs (miRNAs) are small non-coding RNA and negative post-transcriptional regulators of gene expression involved in the modulation of multiple biological processes, including aging [21], and are emerging as promising druggable targets [22]. The microRNA-34a (miR-34a) is an age-associated miRNA since its expression increases in several aged organs and tissues [2,23,24,25]. In the heart, genetic deletion of *Mir34a* reduces the contractile function decline in old mice by inhibiting cardiomyocytes apoptosis [23]. miR-34a is also implicated in endothelial and endothelial progenitor cells acquisition of a senescent phenotype through direct downregulation of its target SIRT1 [26,27].

Our recent work showed that miR-34a is a promoter of senescence-induced VC [28]. miR-34a is upregulated in senescent VSMCs and in aged mouse aortas [2]. Its overexpression in VSMCs promotes osteoblastic differentiation and mineralization by inhibiting proliferation and inducing senescence through direct downregulation of Axl and SIRT1 [2,28]. In vivo, miR-34a is upregulated before aortas calcification and *Mir34a* deficiency reduces the expression of the VC markers SRY (sex-determining region Y)-box 9 (Sox9) and Runx2 and senescence proteins p16 and p21 and, consequently soft tissue and aorta medial calcium deposition [28]. Notably, miR-34a is also able to enhance the transcriptional expression of a subset of SASP molecules [2], however, a direct evidence of miR-34a involvement in arterial inflammaging is still unknown.

In this study, we investigated whether miR-34a influences the production and secretion of SASP factors, in particular IL6, and thus, the spreading of VSMCs mineralization and VC.

Our findings indicate that miR-34a is a central player of arterial inflammaging, since its age-dependent increase in VSMCs enhances senescence and inflammation and, therefore supports arterial dysfunctions such as VC. Hence, miR-34a could represent a promising target to develop a beneficial strategy to favor healthy lifespan.

## 2. Results

### 2.1. miR-34a and IL6 Expression Increase and Correlate During Vascular Aging in Mice and Human Aortic Vascular Smooth Muscle Cells Senescence

**Figure 1:**
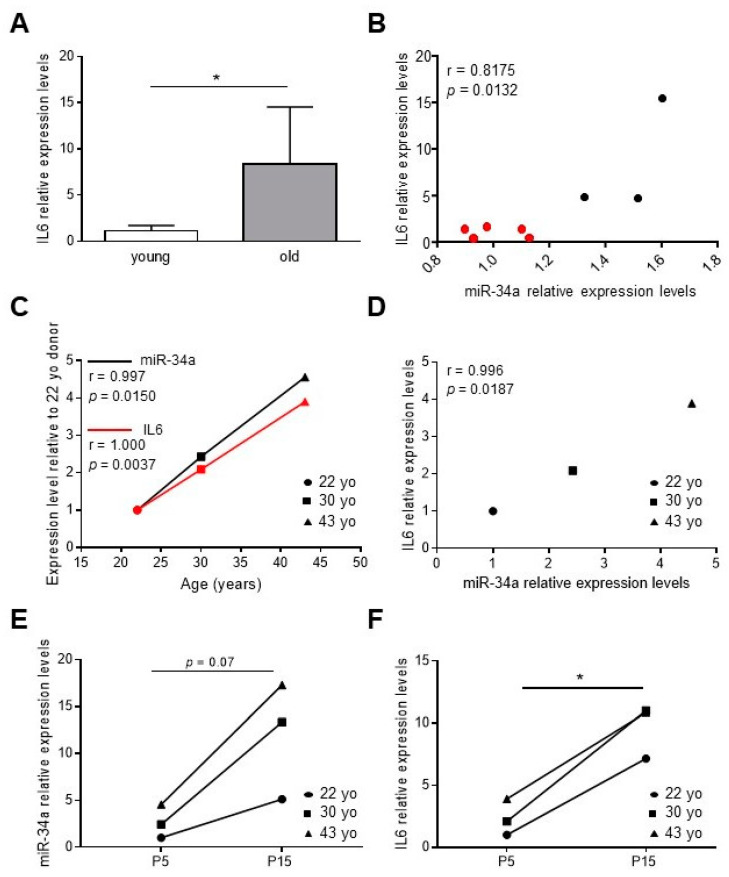
IL6 levels increase and correlate with miR-34a expression during vascular aging and human aortic smooth muscle cells (HASMCs) senescence. (**A**) IL6 expression in aortas of 2.5-month-old (young) and 21-month-old (old) mice analyzed by qRT-PCR and normalized to HPRT levels. Values represent the means ± SD. *, *p* <0.05; Mann Whitney test; *n* = 5 young, 3 old mice. (**B**) Correlation analysis between miR-34a and IL6 levels in mice aortas. r = Pearson’s coefficient; *n* = 5 young (red), 3 old (black) mice. (**C**,**D**) The miR-34a and IL6 mRNA expression in replicative young human aortic smooth muscle cells (HASMCs), isolated from different donors of indicated age (year-old; yo), was evaluated by qRT-PCR and normalized to corresponding U6 and GAPDH levels, respectively. Correlation analysis between (**C**) age (Age; years) and miR-34a or IL6 mRNA levels and (**D**) miR-34a and IL6 mRNA levels. r = Pearson’s coefficient; *n* = 3 donors. (**E**,**F**) miR-34a and IL6 mRNA expression in HASMCs isolated from donors of indicated age (year-old; yo) at young replicative (P5) and senescent (P15) passages evaluated by qRT-PCR and normalized to corresponding U6 and GAPDH levels, respectively. *, *p* <0.05; paired *t* test; *n* = 3 donors.

We have previously demonstrated that miR-34a levels increase in the aortas of aged mice along with senescence markers, such as p16 and p21, and VC-associated proteins, such as Runx2 [2,28]. In order to study whether miR-34a may regulate vascular inflammaging, we measured levels of IL6 in the aorta of young (2.5 months) and old mice (21 months) and correlated with those of miR-34a. IL6 transcript was higher in the aortas of old mice compared to young animals (Figure 1A) and positively correlated with miR-34a expression (r = 0.8175, *p* = 0.0132; Figure 1B).

Then, we measured miR-34a and IL6 levels in human aortic smooth muscle cells (HASMCs) isolated from donors of different ages (22-, 30- and 43-year-old) at proliferative (P5, young) and senescent (P15, old; (Figure S1) [2]) culture passages. Expression of both molecules rose with donor age (Figure 1C; miR-34a, r = 0.9997, *p* = 0.0150; IL6, r = 1.000; *p* = 0.0037) and positively correlated (Figure 1D; r = 0.9996; *p* = 0.0187) and increased with replicative senescence of cells (Figure 1E,F).

These data demonstrate that miR-34a and IL6 amount increase simultaneously during vascular aging in mice and with HASMCs donors’ age and senescence.

### 2.2. miR-34a Induces IL6 Expression and Secretion in HASMCs

In order to investigate if miR-34a directly affects IL6 expression, we transfected HASMCs with a miR-34a mimic (miR-34a) or hairpin inhibitor (anti-miR-34a) and corresponding negative controls (SCR). miR-34a overexpression (Figure S2A) and downregulation (Figure S2B) were able to increase or decrease IL6 mRNA expression, respectively, in all donors compared to corresponding controls.

**Figure 2:**
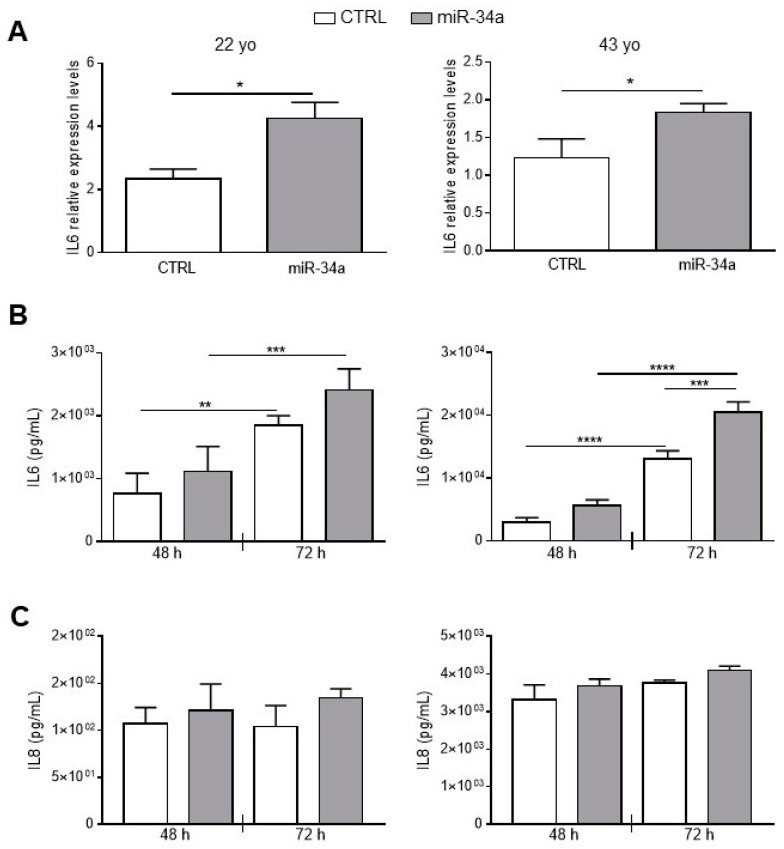
IL6 and IL8 expression and secretion increases upon miR-34a overexpression in HASMCs. HASMCs of 22- or 43-year-old donors were infected with either pMIRNA1 (CTRL) or pMIRH34a (miR-34a) lentivirus. (**A**) HASMCs were cultured in a growth medium for 48 hour. IL6 expression was quantified by qRT-PCR and normalized to corresponding HPRT levels. Values are mean ± SD; *, *p* < 0.05; Student’s *t*-test; *n* = 3–4. (**B**) HASMCs were cultured in growth medium for 48 and 72 hours (h). Amount of IL6 was quantified by ELISA assay in the supernatant of cells. Values are mean ± SD; **, *p* < 0.01; ***, *p* < 0.001; ****, *p* < 0.0001; 1-way ANOVA followed by Bonferroni’s multiple comparison test; *n* = 3–4. (**C**) HASMCs were cultured in growth medium for 48 and 72 h. Amount of IL8 was quantified by ELISA assay in the supernatant of cells. Values are mean ± SD; 22 yo, *n* = 5; 43 yo, *n* = 3.

To better investigate miR-34a and IL6 relationship in HASMCs, we stably overexpressed miR-34a in HASMCs isolated from the 22- and 43-year-old donors by infection with pMIRNA1 (CTRL) or pMIRH34a (miR-34a) lentivirus. As previously published, overexpression of miR-34a in HASMCs increased levels of p21 expression and enhanced senescence-associated β-galactosidase (SA-βgal) activity (Figure S3; [2]). Notably, miR-34a was able to upregulate significantly the mRNA level of IL6 in cells of both donors 48 h after infection (Figure 2A). IL6 was continuously secreted from CTRL HASMCs and overexpression of miR-34a enhanced its production in cells of both donors and in a significant manner in HASMCs of the 43 year-donor (Figure 2B). Notably, IL6 secretion was approximately 10 times higher in HASMCs of the 43- compared to the 22-year-old donor indicating that the production of IL6 increased with aging (Figure 2B).

As a check of the specificity of the effect of miR-34a, we evaluated secretion of Interleukin 8 (IL8). No significant changes were observed in IL-8 production after miR-34a overexpression in HASMCs from the 22- and the 43-year-old donors at 48 and 72 h after infection (Figure 2C). Similarly to IL6, IL8 levels were approximately 10 times higher in HASMCs from the 43- compared to the 22-year-old donor (Figure 2C) confirming that the inflammatory phenotype of HASMCs rises with aging.

These results show that miR-34a specifically modulated expression and secretion of IL6 in HASMCs.

### 2.3. The Secretome of miR-34a-Overexpressing HASMCs Accelerates Cell Calcification

**Figure 3:**
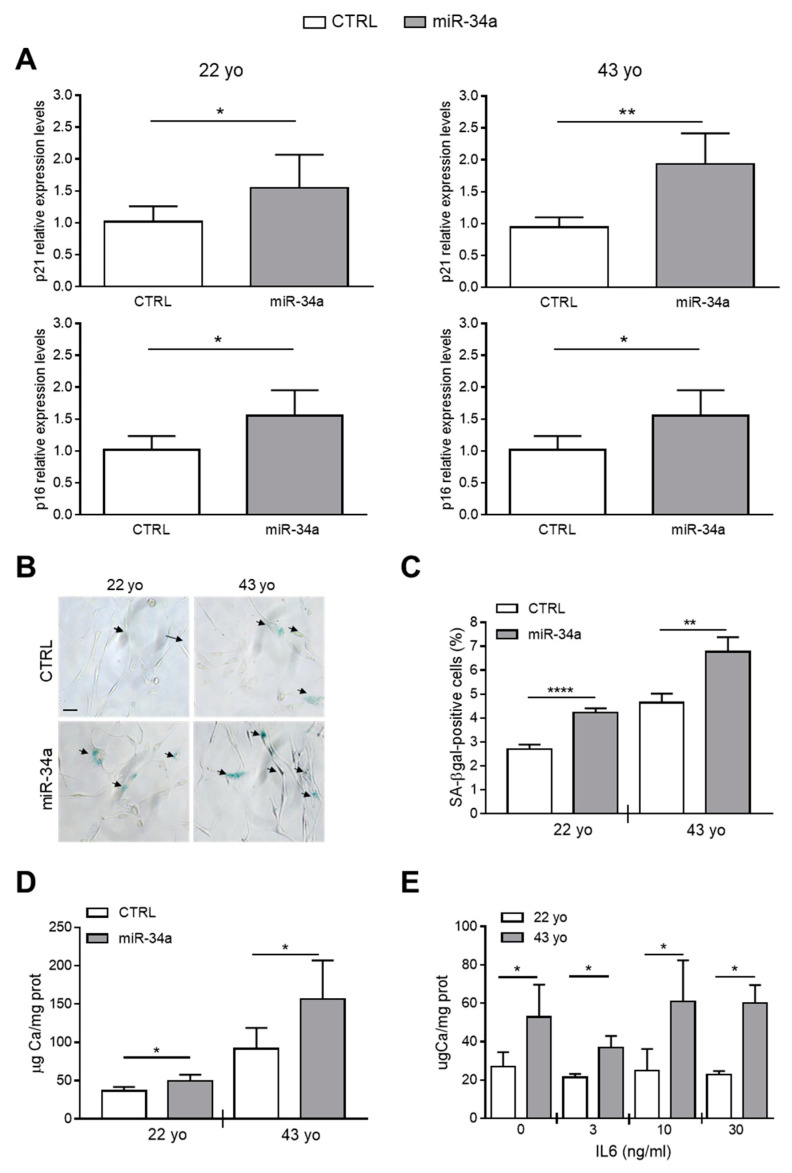
Conditioned medium of miR-34a-overexpressing HASMCs enhances their senescence and calcification. Conditioned medium of HASMCs from 22- or 43-year-old (yo) donors was collected 72 h after infection with pMIRNA1 (CTRL) or pMIRH34a (miR-34a) lentivirus. (**A**) HASMCs were cultured for 24 h in the presence of corresponding conditioned medium. p21 and p16 expression was quantified by qRT-PCR and normalized to corresponding HPRT levels. Values are mean ± SD; *, *p* < 0.05; **, *p* < 0.01; Student’s *t*-test (22 yo); Mann–Whitney test (43 yo); *n* = 6. (**B**,**C**) HASMCs were cultured for 24 h in the presence of corresponding conditioned medium and processed for senescence-associated β-galactosidase (SA-β-gal) staining. (**B**) Representative images of SA-β-gal staining. Bar = 100 µm. **(C)** Bars show quantification of SA-β-gal-positive cells relative to (B). Values are mean ± SD; **, *p* < 0.01; ****, *p* < 0.0001; Student’s *t*-test; 22 yo, *n* = 3; 43 yo, *n* = 4. (**D**) Cells were cultured for 24 h in the presence of the corresponding conditioned medium and then in the osteogenic medium for 7 days. Calcification was measured by colorimetric analysis. Values are mean ± SD; *, *p* < 0.05; **, *p* < 0.01; ****, *p* < 0.0001; Student’s *t*-test; 22 yo, *n* = 6, 6; 43 yo, *n* = 6, 4. (**E**) HASMCs of 22- and 43 year-old (yo) donors were pretreated with indicated concentration of recombinant IL6 and subsequently cultured in osteogenic medium for 7 days. Calcification was measured by colorimetric analysis. Values are mean ± SD; *, *p* < 0.05; Student’s *t*-test; *n* = 3–4.

Our published data indicate that miR-34a promotes the mineralization of VSMCs because it increases their senescence through Axl and SIRT1 downregulation [2,28]. Now, we investigated whether miR-34a-induced secretome can directly modulate HASMCs senescence and hence, their mineralization. HASMCs from the 22- or 43-year-old donor were cultured for 24 h in the presence of conditioned medium of CTRL or miR-34a-overexpressing corresponding HASMCs and then processed for expression of senescence markers. Levels of p21 and p16 and the percentage of SA-β-gal-positive cells were significantly upregulated after preconditioning with miR-34a-overexpressing supernatants compared to CTRL in both donors (Figure 3A–C). Interestingly, HASMCs cultured in osteogenic medium for 7 days showed a significant increase of calcium deposition when preconditioned with miR-34a-induced secretome (Figure 3D).

Finally, we checked whether pretreatment of HASMCs with IL6 alone is capable of enhancing their mineralization. We pretreated 22- and 43-year-old donor HASMCs for 24 h with increasing concentrations of recombinant IL6 (0-3-10-30 ng/mL) and then cultured them in osteogenic medium for 7 days. Pretreatment with IL6 did not affect the calcification process of HASMCs of both donors that appeared to be greater in cells of the 43- compared to the 22-year-old donor (Figure 3E), likely because it did not induce p21 and p16 expression but only SA-β-gal activity (Figure S4).

Altogether, these data indicate that IL6 preconditioning alone, or at least for the time of treatment that we tested, was not sufficient to promote the HASMCs senescent phenotype able to favor calcification. Conversely, the secretome of miR-34a-overexpressing HASMCs was able to enhance cell senescence and subsequent mineralization suggesting that several secreted SASP factors dependent from miR-34a are necessary for the calcification spreading.

### 2.4. miR-34a Promotes the Secretion of Several SASP Factors in HASMCs

In order to identify the plethora of SASP molecules under miR-34a control and driving the osteogenic differentiation of HASMCs, we analyzed supernatants of the 43-year-old donor cells 72 h after lentivirus infection with either pMIRNA1 (SCR) or pMIRH34a (miR-34a) with a human cytokines array [16]. Of the 80 cytokines present on the array, miR-34a induced a general increase in the release of most factors except for BDNF that instead tended to decrease (Table S1). Of note, miR-34a significantly enhanced the secretion of inflammatory cytokines and chemokines such as IL6, IL12, IL13, the Growth-Regulated Oncogene-alfa (GRO-α), the Monokine-induced by gamma Interferon (MIG), the human cytokine I-309 (I-309), the pulmonary and activation-regulated chemokine (PARC), the Macrophage Inflammatory Proteins-1 delta (MIP-1δ) and the anti-inflammatory cytokine IL10, as well as growth factors such as Insulin-like Growth Factor Binding Protein 3 (IGFBP-3) and the placental growth factor (PIGF) and metalloprotease inhibitor TIMP-2 (Table 1). Hence, several SASP factors, including IL6, are under the influence of miR-34a.

### 2.5. Mir34a Genetic Ablation Reduces IL6 Expression During VC

**Figure 4:**
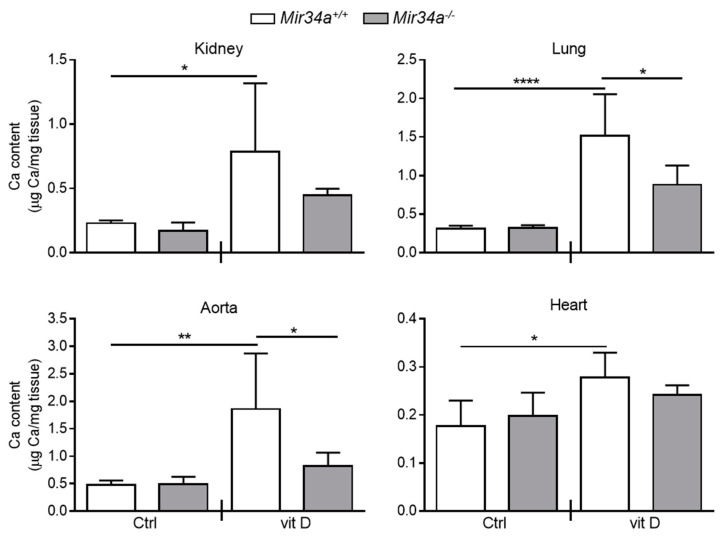
Calcium deposition in *Mir34a^+/+^* and *Mir34a^−/−^* mice after vitamin D treatment. Twelve-week-old *Mir34a^+/+^* and *Mir34a^−/−^* mice were treated subcutaneously with either vitamin D (vit D) or a mock solution (Ctrl) for three consecutive days and sacrificed 5 days (Day 5) after the first injection. Calcium content in kidney, lung, heart and aorta (aortic arch) was quantified by the colorimetric analysis. Values are mean ± SD; *, *p* < 0.05; **, *p* < 0.01; ****, *p* < 0.0001; 1-way ANOVA followed by Bonferroni’s multiple comparison test; *n* = 5, 5, 4 and 5.

We have previously demonstrated that *Mir34a^−/−^* mice are partially protected from developing soft tissue and aortas medial calcification induced by an overdose of vitamin D [28]. Here, we investigated whether miR-34a is able to modulate IL6 expression in the early stage of VC in vivo. *Mir34a^+\+^* and *Mir34a^−\−^* mice were treated with a toxic dose of vitamin D (vit D) or a placebo (Ctrl) by subcutaneous injection for 3 consecutive days and sacrificed 3 or 5 days (Day 3 and Day 5, respectively) after the first administration. As previously demonstrated, calcium deposits increased in various organs, including the aorta, of vit D-treated *Mir34a^+\+^* mice compared to the corresponding Ctrl group while it did not in the organs of *Mir34a^−\−^* mice treated with vit D on Day 5 (Figure 4). 

**Figure 5:**
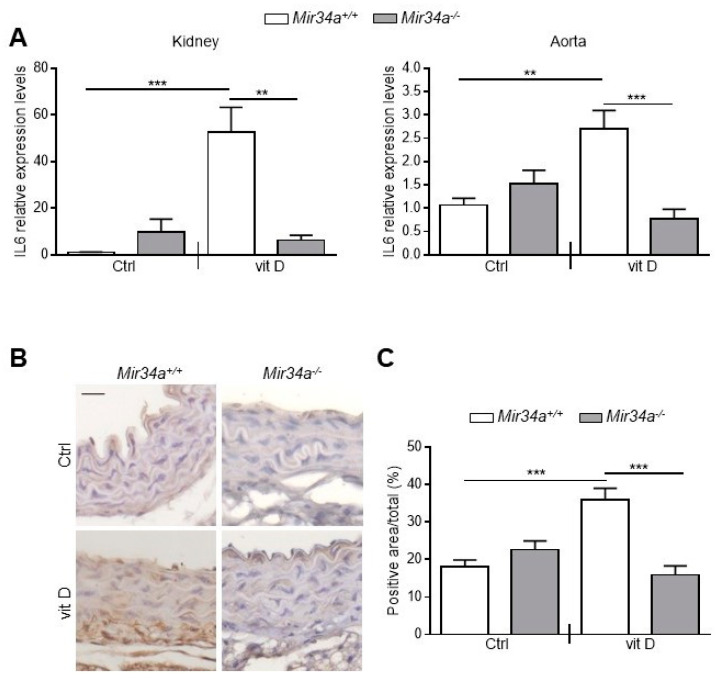
IL6 expression in *Mir34a^+/+^* and *Mir34a^−/−^* mice early after vitamin D treatment. Twelve-week-old *Mir34a^+/+^* and *Mir34a^−/−^* mice were treated subcutaneously with either vitamin D (vit D) or a mock solution (Ctrl) for three consecutive days and sacrificed 3 days after the first injection (Day 3). (**A**) IL6 expression was analyzed by qRT-PCR and normalized to HPRT levels in the kidney and abdominal aorta. Values are mean ± SD. ** *p* < 0.01, *** *p* < 0.001; 1-way ANOVA with a Bonferroni post hoc test; *n* = 6, 5, 6–7 and 4–5. (**B**) Representative images of thoracic aorta sections stained for IL6 expression with a specific antibody. Bar = 20 μm. (**C**) Bars show quantification of the percentage of IL6 positive area to the total thoracic aortic area relative to (**B**). Values are mean ± SD; ***, *p* < 0.001; 1-way ANOVA followed by Bonferroni’s multiple comparison test; *n* = 4, 4, 4 and 3.

Then, we assessed IL6 mRNA levels at Day 3 in the kidney and aorta before the onset of an overt calcification [28]. We found that IL6 expression rose in both organs of vit D-treated *Mir34a^+\+^* but not *Mir34a^−\−^* mice (Figure 5A). Finally, we analyzed by immunohistochemistry the IL6 protein expression pattern. IL6 was induced in VSMCs and endothelial cells of aortas of *Mir34a^+/+^* mice treated with vit D compared to the *Mir34a^+/+^* Ctrl group on Day 3 (Figure 5B,C) and remained high on Day 5 (Figure S5A,B). Notably, administration of vit D did not induce IL6 expression in *Mir34^−/−^* animals on both days (Figure 5B,C; Figure S5A,B).

Altogether, these data indicate that IL6 levels increase before the development of tissue calcification and suggest that the genetic ablation of *Mir34a* protects against arterial inflammation and VC onset likely by preventing IL6 enhancement.

### 2.6. Circulating miR-34a Correlates with IL6 and Not with IL8 Levels in a Healthy Population

**Figure 6:**
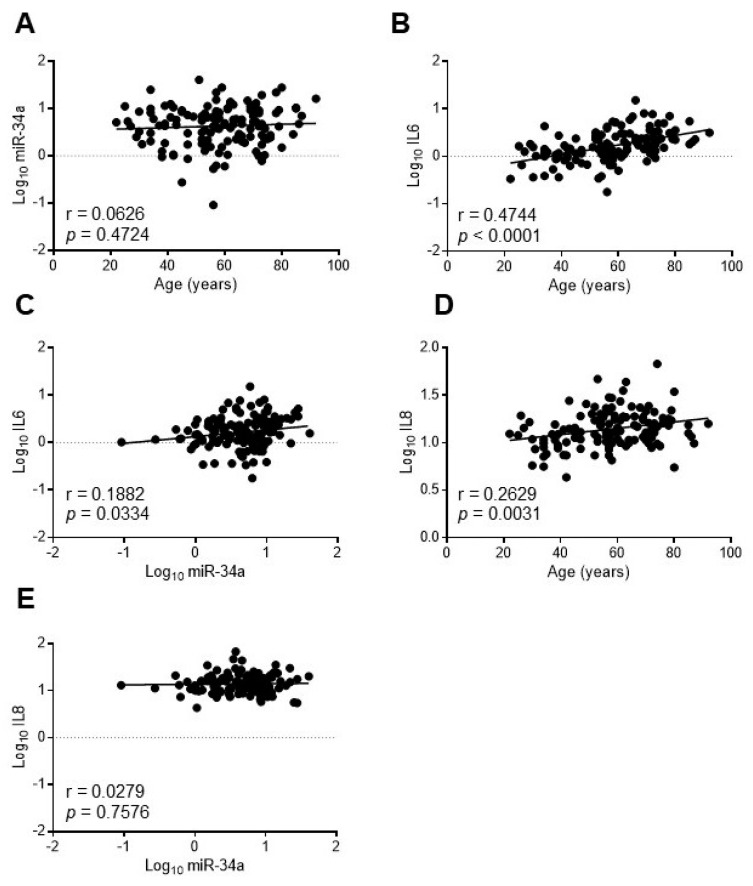
IL6 but not IL8 levels correlate with miR34a expression in healthy subjects. The expression levels of miR-34a were evaluated by qRT-PCR and IL6 or IL8 by ELISA in the serum of 20-90 years-old healthy subjects. Scatter plots showing correlation between age and miR-34a (**A**), age and IL6 (**B**), miR34a and IL6 (**C**), age and IL8 (**D**), miR-34a and IL8 (**E**). Variables showed a skewness distribution and were log-transformed. r = Pearson’s coefficient; *n* = 127–128.

In order to assess whether miR-34a may reflect the systemic inflammaging status, we investigated the relationship between miR-34a and IL6 in the serum of a healthy population of 128 subjects aged 20–90 years old (yo) stratified into three age groups according to the World Health Organization: young (≤45 yo), middle age (46–64 yo) and elderly (≥65 yo) [29]. Population features are reported in Table S2 [30]. As expected IL6 increased progressively across the age groups while miR-34a levels remained almost constant (Table S2). Accordingly, miR-34a did not correlate with age (r = 0.0626, *p* = 0.4724; Figure 6A) whereas IL6 showed a positive correlation (r = 0.4744, *p* < 0.0001; Figure 6B). Notably, miR-34a and IL6 variations showed a significant direct correlation (r = 0.1882, *p* = 0.0334; Figure 6C). Interestingly, IL8 augmented as well across the age groups (Table S2) and positively associated with age (r = 0.2629, *p* = 0.0031; Figure 6D) but did not correlate with miR-34a (r = 0.0279, *p* = 0.7576; Figure 6E).

These results indicate that in a healthy population circulating miR-34a levels, while remaining almost unchanged with advancing age, correlate with IL6 but not with IL8.

## 3. Discussion

Cellular senescence is considered as a trigger of the low-grade, chronic and systemic pro-inflammatory state named inflammaging [10]. The rate of progression of inflammaging is currently recognized not only as the central force driving aging but also as the main risk factors for clinical morbidity and mortality in the elderly [31]. VC is a detrimental complication associated to aging and diseases evincing hallmarks of premature aging such as atherosclerosis, CKD and T2DM and senescent VSMCs and their SASP are pivotal for cell mineralization and VC onset [13,16,20].

We have previously reported that miR-34a increases with vascular aging and is an indirect promoter of VC being necessary for the establishment of VSMCs senescence that, in turn, accelerates the progression of calcification in hyperphosphatemia condition. Indeed, miR-34a levels are high in aged murine aortas and are transiently upregulated in aortas before an overt calcification [2,28]. *Mir34a* ablation protects the mouse from developing soft tissue and aorta medial calcification and in vitro miR-34a promotes senescence-induced VSMCs osteoblastic transdifferentiation and mineralization through downregulation of its targets Axl and SIRT1, which exert protective activities against VC [28]. Interestingly, in the present study we further demonstrate that miR-34a is able to endorse the activation of the SASP in VSMCs thus, fueling the inflammatory condition that drives the spreading of vascular senescence and eventually, calcification progression.

We mainly focused on the relationship between miR-34a and the prototypical pro-inflammatory SASP component IL6, which is canonically upregulated in cells that have undergone senescence, increases with age and plays a causal role in inflammaging and age-related diseases [8,30,32,33]. Besides, IL6 is one of the most abundant cytokines produced by senescent VSMCs and exerts pro-calcification activity [8,34,35]. We found that miR-34a and IL6 levels are upregulated and positively correlate in aortas of old mice and with HASMCs donors’ age and senescence (Figure 1). Notably, miR-34a directly modulates IL6 in HASMCs and its overexpression enhances IL6 mRNA levels and protein secretion (Figure 2A,B). Conversely, miR-34a does not influence IL8 secretion amount (Figure 2C). Basal and miR-34a-induced IL6 and IL8 release rose with HASMCs donor age, which associates with a higher propensity of cells to calcify as well (Figure 2 and Figure 3E). Likely, this is ascribable to the higher percentage of senescent cells present in the culture of the oldest donor [28].

Notably, miR-34a enhances the basal secretion of several other molecules already recognized as SASP factors in HASMCs ([16]; Table 1). Among them, we found mostly pro-inflammatory cytokines and chemokines (i.e., IL12, IL13 and GRO-α; [36]), the protease inhibitor TIMP2, involved in the extracellular matrix remodeling [37], and the growth factor IGFBP3 that is known to be secreted upon replicative or stress-mediated senescence from various primary cell types and to promote senescence of neighboring cells [37,38,39,40]. Interestingly, preconditioning with miR-34a-induced secretome boosts HASMCs senescence and eventually mineralization (Figure 3A–D) and *Mir34a* genetic deletion prevents the increase of IL6 expression occurring in VSMCs during aortas medial calcification onset in vivo (Figure 4 and Figure 5). Hence, miR-34a-induced SASP factors include molecules involved in a paracrine circuit that promotes dissemination of VSMCs inflammation and senescence, conditions that favor their switching to an osteoblastic phenotype in a pro-calcification milieu. The specific relationship between miR-34a-dependent SASP molecules and VC requires additional studies.

The mechanisms by which miR-34a regulates SASP acquisition in VSMCs is currently unknown. SIRT1 downregulation and NF-κB activation have been implicated in SASP induction [6,41]; moreover, it has been demonstrated that VSMCs with persistent DNA damage develop a specific SASP [16]. Determining whether miR-34a can act through these pathways needs further investigations.

Senescent VSMCs and endothelial cells are key players in the induction and maintenance of arterial and systemic inflammaging [12] and the vascular age-associated miR-34a increase may contribute to both. Accordingly, we found a positive correlation between circulating miR-34a and IL6 but not IL8 (confirming the in vitro data on HASMCs; Figure 2C) in healthy subjects of 20–90 years (Figure 6C–E), reinforcing the hypothesis that miR-34a could be a functional promoter of vascular aging and consequent systemic low-grade inflammation. miR-34a has been also shown to control age-related endothelial cells dysfunction [42]. Indeed, miR-34a increases in aged primary endothelial cells and mediates endothelial cell senescence through SIRT1 inactivation [26]. Moreover, miR-34a inhibition improves hyperglycemia-induced aortic endothelial inflammation and senescence [43]. Thus, our present data expand previously published reports supporting the notion that miR-34a may act at multiple levels and by modulating senescence and inflammation of the two most abundant cell types present in the vessel wall, namely endothelial cells and VSMCs, represents a key link between cellular senescence, vascular aging and systemic inflammaging.

In conclusion, aging of the vascular wall is associated with a higher frequency of pathologies and is considered as an important predictor of individual risk of developing cardiovascular diseases. In this framework, miR-34a could represent both an innovative biomarker of vascular aging/inflammaging and a promising target for an effective strategy against age-related vascular complications, including VC.

## 4. Materials and Methods

An expanded methods section is provided in the online Supplementary Materials.

### 4.1. Cell Transfection and Lentiviral Infection

HASMCs were purchased from Lonza (Basel, Switzerland) and cultured in SmGM-2 medium (Lonza). The donors were Caucasian males of 22, 30 and 43 year-old. HASMCs (7.9 × 10^3^ cells/cm^2^) at passage 5–7 were transfected with the miRIDIAN hsa-miR-34a Mimic or the miRIDIAN microRNA Mimic Control or with the miRIDIAN hsa-miR-34a Hairpin Inhibitor or the miRIDIAN microRNA Hairpin Inhibitor Control (Thermo Scientific Dharmacon, Lafayette, CO, USA) using the siRNA Transfection Reagent (Santa Cruz Biotechnology, Santa Cruz, CA, USA) [2]. Cells were processed for miR-34a and IL6 mRNA expression.

The lentiviral vector expressing miR-34a (pMIRH34a) and the control vector (pMIRNA1; CTRL) were purchased from System Biosciences (SBI, Palo Alto, CA, USA). Lentiviruses were produced as in [28] and added to HASMCs at a MOI of 10–20. Briefly, 1.58 × 10^4^ cells/cm^2^ were infected with pMIRH34a or pMIRNA1 viruses. After 24 h, the medium was replaced. Supernatants analysis and RNA extraction were performed 48 and/or 72 h later.

### 4.2. Calcification Assay

After lentiviral infection, HASMCs were cultured in osteogenic medium (DMEM with 15% FBS, 5 mM phosphate, 10 mM sodium pyruvate and 50 ug/mL ascorbic acid) for 37 days. For preconditioned experiments, HASMCs were pretreated with increasing concentration (0, 3, 10 and 30 ng/mL) of recombinant IL6 (Bio-techne Srl. Milan, Italy) or conditioned medium collected 72 h after infection and, then cultured in an osteogenic medium for 7 days.

To quantify the precipitated calcium (expressed as µg Ca/mg proteins), cells were processed as described in [28] with the QuantiChrom™ Calcium Assay Kit (DICA-500, Gentaur, Kampenhout, Belgium). Protein concentration was determined with the Bio-Rad assay (Bio-Rad Laboratories, Hercules, CA, USA).

### 4.3. ELISA Assay

Supernatant from HASMCs was collected 48 and 72 h after infection, centrifuged at 12,000× *g* for 10 min, transferred into a polypropylene tubes and stored at −80 °C. ELISA kits specific for IL6, (DuoSet^®^ cat N°DY206-05, R&D Systems, Minneapolis, MN, USA) and IL8 (DuoSet^®^ cat N°DY208-05, R&D Systems) were used following the manufacturer’s instruction.

### 4.4. Cytokine Arrays

A human cytokine antibody array (ab133998, Abcam, Cambridge, United Kingdom) was used to analyze the conditioned medium of HASMCs infected with lentiviral vector expressing miR-34a (pMIRH34a) or the control empty vector (pMIRNA1; CTRL). All steps for the analysis were performed according to the manufacturer’s instructions. Briefly, 5.3 × 10^3^ cells/cm^2^ at passage 7 were seeded and the next day infected with pMIRH34a or pMIRNA1 viruses. The medium was replaced after 24 h and supernatants were collected 72 h later. Membranes were incubated at 4 °C overnight with 750 µL of the HASMCs conditioned medium. One of the membranes was incubated with cultured medium alone as a sample “blank”. Chemiluminescence reaction was detected using the supplied detection buffers and acquired with a ChemiDoc™ MP Imaging System (Biorad, Hercules, CA, USA). Densitometry data were obtained using ImageJ software. Data were normalized following the manufacturer’s instructions and represented as miR-34a/CTRL ratio.

### 4.5. Animal Experiments

Animal procedures were performed in conformity with the guidelines from the Directive 2010/63/EU of the European Parliament on the protection of animals used for scientific purposes and in accordance with experimental protocols approved by the Committee on Animal Resources at the University of Milan and/or Cogentech (734-2015; approved on 17/07/2015). JAX™ C57BL/6J mice (*Mir34a^+/+^*, wild-type) were purchased from Charles River Laboratories International, Inc. (Stock No: 000664; Wilmington, MA, USA). *Mir34a^−/−^* mouse line was purchased from The Jackson Laboratory (Stock No: 018279; Bar Harbor, ME, USA).

Twelve-week-old male *Mir34a^−/−^* and *Mir34a^+/+^* were treated with either 500,000 IU/kg/day vitamin D (Cholecalciferolor, C1357, Sigma-Aldrich, St. Louis, MO, USA) or a mock solution (1% (*v*/*v*) ethanol, 7% (*v*/*v*) Kolliphor^®^ EL and 3.75% (*w*/*v*) dextrose (Sigma-Aldrich)) administrated subcutaneously for three consecutive days and sacrificed five days after the first injection [28]. Animals were anesthetized with an intraperitoneal injection of ketamine:medetomidine cocktail (100 mg/Kg:10 mg/Kg) and perfused with PBS. Aortas, hearts, lungs and kidneys were processed as described below.

For the aging experiment, aortas were isolated from C57BL/6J male young (2.5-month-old) and old (21-month-old) mice and immediately frozen for RNA extraction.

### 4.6. Tissue Calcium Content Quantification

Dissected organs were washed in PBS, carefully blotted dry, weighted and incubated at 4 °C for 24 h in 20 µL/mg dry weight of 0.6 N HCl. Calcium amount was quantified with QuantiChrom™ Calcium Assay Kit (Gentaur) following the manufacturer’s protocol and normalized to the dry tissue weight (µg Ca/mg tissue).

### 4.7. Quantitative RT-PCR (q-RT-PCR)

Total RNA from HASMCs or murine organs was extracted using TRIzol reagent (Invitrogen, Carlsbad, CA, USA) or miRNeasy Mini Kit (#217004, QIAGEN, Hilden, Germany), respectively, following the manufacturer’s protocol. Gene expression levels were determined using the 2^−ΔΔCT^ method; *HPRT* and *GAPDH* were used as reference for human genes while snoRNA202 and U6 for mouse and human miR-34a, respectively (Table S3).

### 4.8. Immunohistochemistry

Mouse distal thoracic aortas were fixed in 10% formalin and paraffin embedded. Six micrometer sections were boiled for 20 min in Dako Target Retrieval Solution Citrate pH 9 (Aligent Technologies, Santa Clara, CA, USA) and then blocked in 5% goat serum in PBS-T for 45 min at room temperature. Primary antibody against IL6 (5 μg/mL, AF-406-NA, R&D Systems) was dissolved in 1% goat serum PBS-T and incubated overnight at 4 °C in a humidified chamber. Sections were counterstained with hematoxylin. Images were acquired with an Axioskop II microscope (Zeiss, Oberkochen, Germany) using a digital camera (AxioCam Color, Zeiss).

The quantification of IL6 signal was carried after acquiring the images with an Axioskop II microscope (Zeiss) using a digital camera (AxioCam Color, Zeiss) on the entire aorta cross section with the Axiovision Software Rel 4.7 (Zeiss). The percentage of positive area was defined as the ratio between IL6 positive area to the total area of the aortas.

### 4.9. Human Study

Participants were recruited from the Italian National Research Center on Aging (INRCA), Ancona [30]. All subjects (128 healthy subjects aged 20–90 years (M = 61, F = 67)) gave their written informed consent to participate in the study, which was approved by INRCA’s Ethics Committee. The health status was assessed using standardized questionnaires, laboratory assays and physical examination. An ELISA kit was used to test IL6 (#HS600B, R&D Systems Inc.) and IL8 (#HS800; R&D Systems Inc.) serum levels following the manufacturer’s instruction.

For miR-34a detection, total RNA was isolated from 50 μL of serum with the Total RNA Purification Kit (Norgen Biotek Corporation, Thorold, ON, Canada) according to the manufacturer’s protocol. Synthetic *C. elegans* cel-miR-39 was added before RNA extraction to measure RNA recovery. Real-time PCR for miR-34a was performed with TaqMan miRNA assays (Applied Biosystems, Foster City, CA, USA) run on the CFX96 Touch™ Real-Time PCR Detection System (Bio-Rad Laboratories). The expression of miR-34a relative to cel-miR-39 was determined using the 2^−ΔΔCT^ method.

### 4.10. Statistical Analysis

Data were analyzed with GraphPad Prism software version 7 (GraphPad Software, Inc., La Jolla, CA, USA) or SAS9.4 program. For the human population, qualitative variables were reported as frequencies and percentages. Quantitative variables were reported as mean plus standard deviation (continuous variables normally distributed) or median and interquartile range (continuous variables skewed distributed). Skewed distributed variables were transformed in logarithm to base 10. Population features changes were classified according to age into three classes, young (≤45 yrs), middle age (46–64 yrs) and elderly (≥65 yrs) and linear trends across categories were evaluated. The crude relation between miR-34a and IL6 or IL8 was assessed by Pearson correlation.

For in vitro experiments, the Shapiro–Wilk test was used to assess the normality of distribution of investigated parameters. Differences between two groups were analyzed with a Student’s *t*-test and Mann–Whitney U test for normally and not normally distributed variables, respectively, or a paired *t*-test as stated in figure legends. Statistical analysis between more than two groups was conducted by one-way ANOVA with a Bonferroni post-hoc test. Values are presented as mean ± SD. *p* < 0.05 was considered significant.

## Figures and Tables

**Table 1:** Senescence-associated secretory phenotype (SASP) factors significantly induced by overexpression of miR-34a in HASMCs.

SASP Factors	
Interleukins/chemokines	GRO-α, I-309, IL6, IL10, IL12, IL13, PARC, MIP-1δ
Growth factors/regulators	IGFBP-3, PIGF
Metallopeptidase inhibitor	TIMP-2

GRO-alfa = Growth-Regulated Oncogene-alfa; I-309 = human cytokine I-309; IL6 = Interleukin 6; IL10 = Interleukin 10; IL12 = Interleukin 12; IL13 = Interleukin 13; MIG = Monokine Induced by gamma Interferon; MIP-1 δ = Macrophage Inflammatory Proteins-1 delta; IGFBP-3 = Insulin-like Growth Factor Binding Protein 3; PARC = Pulmonary and activation-regulated chemokine; PIGF = Placental growth factor; TIMP-2 = Metallopeptidase Inhibitor 2.

## Supplementary Materials

The following are available online at https://www.mdpi.com/1422-0067/21/12/4454/s1, Figure S1: Levels of replicative senescence markers in HASMCs isolated from donors of different age., Figure S2: miR-34a modulates IL6 expression, Figure S3: Levels of replicative senescence markers increase upon mi-R34a overexpression in HASMCs, Figure S4: IL6 treatment partially affects HASMCs senescence, Figure S5: IL6 expression in Mir34a+/+ and Mir34a-/- mice after vitamin D treatment, Table S1: The overexpression of miR-34a in HASMCs induces the expression of several SASP factors, Table S2: Biochemical, metabolic and anthropometric variables of healthy subjects divided in three age groups, Table S3: List of primers used for quantitative RT-PCR.
