# Supplementary material for: The microRNA-34a-Induced Senescence-Associated Secretory Phenotype (SASP) Favors Vascular Smooth Muscle Cells Calcification
Source: Int J Mol Sci. 2020 Jun 23;21(12):4454. doi: 10.3390/ijms21124454 (PMC7352675; doi:10.3390/ijms21124454)
Supplement: Supplementary file 1 [file ijms-21-04454-s001.zip › Supplementary Materials/Supplementary Materials.docx]

**The microRNA-34a-induced senescence-associated secretory phenotype (SASP) favors Vascular Smooth Muscle Cells calcification**

Estella Zuccolo^1^, Ileana Badi^1,§^, Francesco Scavello^1^, Irene Gambuzza^1^, Luigi Mancinelli^1, §§^, Federica Macrì^1^, Calogero C. Tedesco^2^, Fabrizio Veglia^2^, Anna Rita Bonfigli^3^, Fabiola Olivieri^4, 5^, Angela Raucci^1,#^

1 Unit of Experimental Cardio-Oncology and Cardiovascular Aging, Centro Cardiologico Monzino-IRCCS, 20138, Milan, Italy. 2 Unit of Biostatistics, Centro Cardiologico Monzino-IRCCS, 20138, Milan, Italy.3 Scientific Direction, IRCCS INRCA, Ancona, Italy. 4 Department of Clinical and Molecular Sciences, DISCLIMO, Università Politecnica delle Marche, Ancona, Italy. 5 Center of Clinical Pathology and Innovative Therapy, IRCCS INRCA, Ancona, Italy.

§ Division of Cardiovascular Medicine, Radcliffe Department of Medicine, University of Oxford, United Kingdom.

§§ Department of Periodontics and Preventive Dentistry, University of Pittsburgh, School of Dental Medicine, Pittsburgh, PA, United States.

**Supplementary MATERIALS**

**MATERIALS and METHODS**

**Cell culture**

Human aortic smooth muscle cells (HASMCs) were purchased from Lonza (Basel, Switzerland) and cultured in SmGM-2 complete medium (Lonza). The donors were Caucasian males of 22, 30 and 43 years.

**Cell transfections and lentiviral infection**

HASMCs at passage 5-7 were transfected with the miRIDIAN hsa-miR-34a Mimic or the miRIDIAN microRNA Mimic Negative Control #1 or with the miRIDIAN hsa-miR-34a Hairpin Inhibitor or the miRIDIAN microRNA Hairpin Inhibitor Negative Control #1 (Thermo Scientific Dharmacon, Lafayette, CO, USA) using the siRNA Transfection Reagent (Santa Cruz Biotechnology, Santa Cruz, CA, USA) according to the manufacturer’s protocol. Briefly, 7.9*10^3^ cells/cm^2^ were seeded and cultured in complete medium overnight. The siRNA Transfection Reagent and the microRNA mimic were diluted in opti-MEM medium (Thermo Fisher Scientific, Waltham, Massachusetts, USA) and incubated 30 minutes at room temperature. After changing the SmGM-2 with optiMEM medium, the nucleic acid-transfection reagent mixture was added to each well to a final microRNA mimic/hairpin inhibitor concentration of 100 nM. After 5 hours, medium was completely replaced with SmGM-2 and the cells were cultured for additional 48 hours [1]. RNA extracts were processed for miR-34a and IL6 expression levels.

The lentiviral vector expressing miR-34a (pMIRH34a) and the control empty vector (pMIRNA1) were purchased from System Biosciences (SBI, Palo Alto, CA, USA). Lentiviruses were produced using 293T cells transfected with a standard calcium phosphate protocol and added to HASMCs at a MOI of 10-20. Briefly, 1.58*10^4^ cells/cm^2^ at passage 7 were seeded and the next day infected with pMIRH34a or pMIRNA1 viruses [2]. The medium was replaced after 24 hours and supernatant analysis and RNA extraction were performed 48 and/or 72 hours after infection as indicated in the figure legends. For calcification experiments, infected cells were cultured in osteogenic medium.

**Senescence-associated β-galactosidase (SA-β-gal) staining**

Senescence was assessed with the SA-β-gal staining kit (Cell Signaling Technology, Danvers, MA, USA) following the manufacturer’s protocol and as described in [1]. Images of ten random fields were acquired using a Zeiss Axiovert 200M microscope equipped with a HITACHI HV-D30 Compact 3-CCD Camera and used to calculate the percentage of SA-β-gal-positive cells over the total number of cells.

**ELISA assay**

Supernatant from HASMCs was collected 48 and 72 hours after infection, centrifuged at 12000 g for 10 minutes, transferred into a polypropylene tubes and stored at -80°C. ELISA kits specific for IL6, (DuoSet® cat n°DY206-05, R&D Systems, Minneapolis, MN, USA) and IL8 (DuoSet® cat n°DY208-05, R&D Systems) were used following manufacturer’s instruction.

**Calcification assay**

HASMCs were cultured in osteogenic medium (DMEM supplemented with 15% FBS, 5 mM phosphate, 10 mM sodium pyruvate and 50 ug/mL ascorbic acid) for 7 days. For pre-conditioned experiments, HASMCs were pretreated with different concentration (0-3-10-30 ng/ml) of recombinant IL6 (Bio-techne Srl.) or conditioned medium collected 72 hours after infection and then cultured in osteogenic medium for 7 days. To quantify the precipitated calcium, cells grown in 12-well plates were quickly washed twice with 500 µL of PBS and incubated overnight with 250 µL of 0.6 N HCl at 4°C; then, supernatants were collected. In order to extract protein for normalization, cells were washed twice with 500 µL of PBS and incubated 4 hours at room temperature with 250 µL of 0.1% SDS-0.1 N NaOH lysis buffer. The precipitated calcium was quantified by colorimetric analysis with the QuantiChrom™ Calcium Assay Kit (DICA-500, Gentaur, Kampenhout, Belgium) whereas and the protein concentration was determined with the Bio-Rad protein assay (Bio-Rad Laboratories, Hercules, CA, USA) and expressed as µg Ca/mg proteins [2].

**Quantitative RT-PCR (q-RT-PCR) on HASMCs**

The total RNA from HASMCs was extracted using TRIzol reagent (Invitrogen, Carlsbad, CA, USA) and treated with the TURBO DNA-free Kit (Invitrogen) or with Illustra RNAspin Mini kit (25-0500-72; GE Healthcare; Chicago, Illinois, USA). cDNA was synthesized with the iScript Reverse Transciption Supermix for RT-qPCR (Bio-Rad Laboratories, Hercules, CA, USA). Real-time PCR was performed on a Bio-Rad iCycler Thermal Cycler with iQ5 Multicolor Real-Time PCR Detection System using the iTaq™ Universal SYBR® Green Supermix (Bio-Rad Laboratories). Relative gene expression levels of IL6, p21 and p16 were determined using the 2^−ΔΔCT^ method; human *HPRT* and *GAPDH* were used as reference genes (Table S3).

**Cytokine Arrays**

A human cytokine antibody array (ab133998, Abcam, Cambridge, United Kingdom) was used to determine relative cytokine levels of HASMCs infected with lentiviral vector expressing miR-34a (pMIRH34a) and the control empty vector (pMIRNA1). The array allows the detection of 80 human cytokines. All steps of the sample analysis procedure were performed according to the manufacturer's instructions. Briefly, 5.3*10^3^ cells/cm^2^ at passage 7 were seeded and the next day infected with pMIRH34a (miR-34a) or pMIRNA1 (CTRL) viruses. After 24 hours, the medium was replaced. Supernatants were collected 72 hours later and used for the analyses. The supplied membranes were blocked with the provided buffer and subsequently incubated at 4°C overnight with 750 µL of the HASMCs supernatant. One of the membranes was incubated with cultured medium alone and used as a sample “blank”. Membranes were washed with the provided buffers and incubated again at 4°C overnight with the supplied biotin-conjugated anti-cytokines. Then, the arrays were washed and incubated with horseradish-peroxidase-conjugated streptavidin for 2 hours at room temperature. Chemiluminescence reaction was detected using the supplied detection buffers and acquired with a ChemiDoc™ MP Imaging System (Biorad, Hercules, CA, USA). Densitometry data were obtained using ImageJ software. Data were normalized using the "blank" array and following the manufacturer's instructions and represented as miR-34a/CTRL ratio.

The membrane can detect the following cytokines: ENA-78, GCSF, GM-CSF, GRO, GRO-alpha (CXCL2), I-309, IL-1alpha, IL-1beta, IL-2, IL-3, IL-4, IL-5, IL-6, IL-7, IL-8 (CXCL8), IL-10, IL-12 p40/p70, IL-13, IL-15, IFN-gamma, MCP-1 (CCL2), MCP-2 (CCL8), MCP-3 (CC7), MCSF, MDC (CCL22), MIG (CXCL9), MIP-1beta (CCL4), MIP-1delta (CCL15), RANTES (CCL5), SCF, SDF-1, TARC (CCL17), TGF-beta1, TNF-alpha, TNF-beta, EGF, IGF-I, Angiogenin, Oncostatin M, Thrombopoietin, VEGF-A, PDGF-BB, Leptin, BDNF, BLC, Ckß8-1, Eotaxin (CCL11), Eotaxin-2 (CCL24), Eotaxin-3 (CCL26), FGF-4, FGF-6, FGF-7, FGF-9, Flt-3 Ligand, Fractalkine, GCP-2, GDNF, HGF, IGFBP-1, IGFBP-2, IGFBP-3, IGFBP-4, IL-16, IP-10 (CXCL10), LIF, LIGHT, MCP-4, MIF, MIP-3 alpha (CCL20), NAP-2 (CXCL7), NT-3, NT-4, Osteopontin, Osteoprotegerin, PARC (CCL18), PLGF, TGF-beta2, TGF-beta3, TIMP-1, TIMP-2.

**Animal experiments**

Animal procedures were performed in conformity with the guidelines from the Directive 2010/63/EU of the European Parliament on the protection of animals used for scientific purposes and in accordance with experimental protocols approved by the Committee on Animal Resources at the University of Milan and/or Cogentech (734-2015; approved on 17/07/2015). Mice were housed in standard cages on a 12:12 h light-dark cycle and fed a normal chow diet *ad libitum*. JAX™ C57BL/6J mice (*Mir34a^+/+^*, wild-type) were purchased from Charles River Laboratories International, Inc. (Stock No: 000664; Wilmington, MA, USA). *Mir34a^-/-^* mouse were purchased from the Jackson Laboratory (Stock No: 018279; Bar Harbor, ME, USA).

Twelve-week-old male *Mir34a^-/-^* and *Mir34a^+/+^* were treated with either 500000 IU/kg/day vitamin D (Cholecalciferolor, C1357, Sigma-Aldrich, St. Louis, MO, USA; ) or a mock solution (1% (v/v) Ethanol, 7% (v/v) Kolliphor^®^ EL, 3.75% (w/v) Dextrose (all from Sigma-Aldrich)) administrated subcutaneously for three consecutive days, and sacrificed five days after the first injection [2]. Animals were anesthetized with an intraperitoneal injection of ketamine:medetomidine cocktail (100mg/Kg:10mg/Kg) and perfused with phosphate-buffered saline (PBS) from the apex of the heart. Aortas, hearts, lungs and kidneys were dissected out, immediately frozen and processed for calcium content or Immunohistochemistry as described below. Abdominal aortas and kidneys were processed for RNA extraction and IL6 mRNA expression.

For the aging experiment, aortas were isolated from C57BL/6J male young (2.5-month-old) and old (21-month-old) mice and immediately frozen for RNA extraction.

**Quantitative RT-PCR (q-RT-PCR) on murine tissues**

Total RNA from murine aortas or kidneys was extracted using miRNeasy Mini Kit (#217004, QIAGEN, Hilden, Germany) following the manufacturer’s protocol. Real-time PCR (qRT-PCR) to detect miR-34a levels was performed with TaqMan miRNA assays (Applied Biosystems) run on the Bio-Rad iCycler Thermal Cycler with iQ5 Multicolor Real-Time PCR Detection System. The expression of miR-34a or IL6 was determined using the 2^−ΔΔCT^ method. snoRNA202 were used as reference genes for mouse miR-34a while *hprt* for mouse IL6 (Table S3).

**Tissue calcium content quantification**

Dissected organs were washed in PBS, carefully blotted dry, weighted and incubated at 4°C for 24 hours in 20 µL/mg dry weight of 0.6 N HCl. The amount of calcium was quantified by a colorimetric analysis with QuantiChrom™ Calcium Assay Kit (Gentaur) following the manufacturer’s protocol. Calcium extracted from the organs was normalized to the tissue dry weight (µg Ca/mg tissue).

**Immunohistochemistry**

Mouse distal thoracic aortas were fixed in 10% formalin and paraffin embedded. Six μm sections were de-paraffinized, re-hydrated and boiled for 20 minutes in Dako Target Retrieval Solution Citrate pH 9 (Aligent Technologies, Santa Clara, CA, USA). After washing in PBS-0.1% Triton X-100 (PBS-T) slides were incubated in 3% H_2_O_2_ (Sigma-Aldrich) for 10 min, to inactivate endogenous peroxidase, and then blocked in 5% goat serum in PBS-T for 45 minutes at room temperature. Primary antibody against IL6 (5 μg/mL, AF-406-NA, R&D Systems) was dissolved in 1% goat serum PBS-T and incubated overnight at 4°C in a humidified chamber. A negative control in which the tissue was incubated with 1% goat serum PBS-T without the primary antibody was also included. The, sections were incubated with biotin-conjugated goat anti-rabbit antibody (1:200, Vector Laboratories, Burlingame, CA, USA) and, then with horseradish peroxidase (HRP)-conjugated streptavidin (ABC kit; PK-6100, Vector Laboratories) for 30 min at room temperature. Immunoreactions were revealed using 3.3'-Diaminobenzidine (ImmPACT DAB substrate, SK-4105, Vector Laboratories) as chromogen and slides were counterstained with hematoxylin. Images were acquired with an Axioskop II microscope (Zeiss, Oberkochen,Germany) using a digital camera (AxioCam Color, Zeiss).

The quantification of IL6 signal was carried after acquiring the images with an Axioskop II microscope (Zeiss) using a digital camera (AxioCam Color, Zeiss) on the entire aorta cross section with the Axiovision Software Rel 4.7 (Zeiss). The percentage of positive area was defined as the ratio between IL6 positive area to the total area of the aortas.

**Human study**

Participants were recruited from the Italian National Research Center on Aging (INRCA), Ancona as described in [3]. All subjects gave their written informed consent to participate in the study, which was approved by INRCA’s Ethics Committee. Serum of roughly 128 healthy subjects aged 20-90 years (yrs) (M=61, F=67) was tested for miR-34a. The health status of subjects was assessed using standardized questionnaires, laboratory assays and physical examination. Subjects were considered healthy if at the time of blood collection they did not have any major acute and/or chronic age-related disease such as acute myocardial infarction (AMI), chronic heart failure (CHF), Alzheimer’s disease (AD), type-2 diabetes mellitus (T2DM) or cancer. Subjects with a Cumulative Illness Rating Scale (CIRS) > 2, which indicates a comorbid state, were excluded [4]. All the studied subjects consumed a Mediterranean diet. Subjects were defined as hypertensive when they were under active treatment or when their systolic blood pressure was >140 mmHg and/or their diastolic blood pressure was >90 mmHg, on three different occasions. Body mass index (BMI) was determined as body weight in Kg over height in m^2^. Waist circumference (WC) was measured at the midpoint between the lower margin of the last palpable ribs and the top of the iliac crest using a measuring tape. Hip circumference (H) was measured around the widest portion of the buttocks. For both measurements, the individual was standing and wearing little clothing. The measurements were taken at the end of a normal respiration. W/H was calculated as waist measurement divided by hip measurement.

Overnight fasting venous blood samples of all subjects were collected from 8:00 to 10:00 a.m. in EDTA and citrate tubes. White blood cell, monocyte and platelet counts were performed by standard automated procedures (Sysmex XE-2100, Kobe, Japan). Blood concentrations of glycosylated hemoglobin (HbA1c) were measured by a G8 HPLC analyzer (TOSOH BIOSCIENCE, USA). An immunoenzymatic method was used for PAI-1 antigen (Biopool, Sweden). Total and HDL cholesterol, fasting insulin, fibrinogen, and apolipoprotein AI and B (ApoAI and ApoB), triglycerides, creatinine, and fasting glucose were measured using commercially available kits on an automated clinical chemistry COBAS analyzer (Roche-Hitachi, Basel, Switzerland). Highly sensitive C-reactive protein (CRP) was determined by the particle-enhanced immunoturbidimetric assay (CRP High Sensitive, Roche-Hitachi) on a COBAS analyzer. HOMA index was calculated as Glucose (mg/100 ml)* Insulin (uIU/ml)/405.

Whole human peripheral blood was collected in a tube without anticoagulants and left to clot undisturbed at room temperature for 15–30 minutes. The clot was removed by centrifuging at 2000 g for 10 minutes at 4°C. The resulting supernatant was immediately transferred into a clean polypropylene tubes, aliquoted and stored at -80°C. An ELISA kit was used to test serum levels of IL6 (#HS600B, R&D Systems Inc., MN, USA) and IL8 (#HS800; R&D Systems Inc.) following manufacturer’s instruction. For miR-34a detection in healthy subjects, total RNA from 50 μL of human serum was isolated with the Total RNA Purification Kit (Norgen Biotek Corporation, Thorold, ON, Canada) according to the manufacturer's protocol. Synthetic C. elegans cel-miR-39 was added before RNA extraction into all samples for the measure of the RNA recovery. Real-time PCR for miR-34a was performed with TaqMan miRNA assays (Applied Biosystems, Foster City, CA, USA) run on the CFX96 Touch™ Real-Time PCR Detection System (Bio-Rad Laboratories, Hercules, CA, USA). The expression of miR-34a relative to cel-miR-39 was determined using the 2^−ΔΔCT^ method.

**Statistical analysis**

Data were analyzed with GraphPad Prism software version 7 (GraphPad Software, Inc, La Jolla, CA, USA) or SAS9.4 program. For the human population, qualitative variables were reported as frequencies and percentages. Quantitative variables were reported as mean plus standard deviation (continuous variables normally distributed) or median and interquartile range (continuous variables skewed distributed). Skewed distributed variables were transformed in logarithm to the base 10. Population features changes were classified according to age into three classes, young (≤45 yrs), middle age (46-64 yrs) and elderly-old (≥65 yrs) and linear trends across categories were evaluated. The crude relation between miR-34a and IL6 or IL8 was assessed by Pearson correlation.

For *in vitro* experiments, the Shapiro-Wilk test was used to assess the normality of distribution of investigated parameters. Differences between two groups were analysed with unpaired Student's t-test or Mann–Whitney U test for normally or not normally distributed variables, respectively, or paired t test as described in figure legends. Statistical analysis between more than two groups was conducted by one-way ANOVA with Bonferroni post-hoc test. Values are presented as mean ± SD. A value of P < 0.05 was considered statistically significant.

**Supplementary Figure Legends**

**Figure S1. Levels of replicative senescence markers in HASMCs isolated from donors of different age. (A, B)** p21 and p16 mRNA expression in young replicative (P5) and old senescent (P15) HASMCs, isolated from donors of indicated age (year-old; yo), was evaluated by qRT-PCR and normalized to corresponding HPRT levels. *, P <0.05; Paired t test; n = 3 donors. **(C)** Representative images of senescence-associated β-galactosidase (SA-β-gal) staining of young replicative (P5) and old senescent (P15) HASMCs isolated from 22- and 43- year-old (yo) donors. Bar = 50 µm. **(D)** Bars represent the percentages of SA-β-gal-positive cells relative to (C). ****, P ≤0.0001; 1-way ANOVA followed by Bonferroni’s multiple comparison test; n= 4, 4, 3, 4.

**Figure S2. miR-34a modulates IL6 expression. (A)** HASMCs of indicated different age donors were transfected with a miR-34a mimic (miR-34a) or a mimic negative control (SCR) and cultured for 48 hours. IL6 expression was quantified by qRT-PCR and normalized to corresponding GAPDH levels. Values are mean ± SD; *, P < 0.05; **, P < 0.01; Student’s t-test; n = 3. **(B)** HASMCs of indicated different age donors were transfected with a miR-34a hairpin inhibitor (anti-miR-34a) or a hairpin inhibitor negative control (SCR) and cultured for 48 hours. IL6 expression was quantified by qRT-PCR and normalized to corresponding GAPDH levels. Values are mean ± SD; *, P < 0.05; Student’s t-test; n ≥ 2.

**Figure S3. Levels of replicative senescence markers increase upon mi-R34a overexpression in HASMCs.** HASMCs of 22- or 43-year-old (yo) donors were infected with either pMIRNA1 (CTRL) or pMIRH34a (miR-34a) lentivirus. (A) HASMCs were cultured in growth medium for 48 hour. p21 expression was quantified by qRT-PCR and normalized to corresponding HPRT levels. Values are mean ± SD; *, P < 0.05; **, P < 0.01; Student’s t-test; 22 yo, n = 5; 43 yo, n=7. (B, C) HASMCs were cultured in growth medium for 72 hours (h). (B) Representative images of senescence-associated β-galactosidase (SA-β-gal)-stained. Bar = 100 µm. (C) Bars show the quantification of percentage of SA-β-gal-positive cells relative to (B). Values are mean ± SD; *, P< 0.05; **, P < 0.01; Student’s t-test; n = 3.

**Figure S4. IL6 treatment partially affects HASMCs senescence.** HASMCs of 22- and 43 year-old (yo) donors were pretreated with 0 or 30 ng/mL of recombinant IL6 for 24 hours. (A) p21 and p16 expression was quantified by qRT-PCR and normalized to corresponding HPRT levels. Values are mean ± SD; 22 yo, n = 8, 7-8; 43 yo, n=7-8, 8. (B, C) HASMCs were processed for senescence-associated β-galactosidase (SA-β-gal) staining. (B) Representative images of SA-β-gal staining. Bar = 100 µm. (C) Bars show quantification of SA-β-gal-positive cells relative to (B). Values are mean ± SD; *, P < 0.05; **, P < 0.01; Student’s t-test; 22 yo, n = 3; 43 yo, n = 3.

**Figure S5. IL6 expression in Mir34a+/+ and Mir34a-/- mice after vitamin D treatment.** Twelve-week-old Mir34a+/+ and Mir34a-/- mice were treated subcutaneously with either vitamin D (vit D) or a mock solution (Ctrl) for three consecutive days and sacrificed 5 days after the first injection (Day 5). (A) Representative images of thoracic aorta sections stained for IL6 expression with a specific antibody. Bar = 20 μm. (B) Bars show quantification of the percentage of IL6 positive area to the total thoracic aortic area relative to (A). Values are mean ± SD; **, P< 0.01; 1-way ANOVA followed by Bonferroni’s multiple comparison test; n= 5, 3, 4, 5.

**REFERENCES**

1. Badi, I.; Burba, I.; Ruggeri, C.; Zeni, F.; Bertolotti, M.; Scopece, A.; Pompilio, G.; Raucci, A. MicroRNA-34a Induces Vascular Smooth Muscle Cells Senescence by SIRT1 Downregulation and Promotes the Expression of Age-Associated Pro-inflammatory Secretory Factors. *J Gerontol A Biol Sci Med Sci* **2015**, *70*, 1304-1311, doi:10.1093/gerona/glu180.

2. Badi, I.; Mancinelli, L.; Polizzotto, A.; Ferri, D.; Zeni, F.; Burba, I.; Milano, G.; Brambilla, F.; Saccu, C.; Bianchi, M.E., et al. miR-34a Promotes Vascular Smooth Muscle Cell Calcification by Downregulating SIRT1 (Sirtuin 1) and Axl (AXL Receptor Tyrosine Kinase). *Arterioscler Thromb Vasc Biol* **2018**, *38*, 2079-2090, doi:10.1161/ATVBAHA.118.311298.

3. Scavello, F.; Zeni, F.; Tedesco, C.C.; Mensa, E.; Veglia, F.; Procopio, A.D.; Bonfigli, A.R.; Olivieri, F.; Raucci, A. Modulation of soluble receptor for advanced glycation end-products (RAGE) isoforms and their ligands in healthy aging. *Aging (Albany NY)* **2019**, *11*, 1648-1663, doi:10.18632/aging.101860.

4. Mistry, R.; Gokhman, I.; Bastani, R.; Gould, R.; Jimenez, E.; Maxwell, A.; McDermott, C.; Rosansky, J.; Van Stone, W.; Jarvik, L., et al. Measuring medical burden using CIRS in older veterans enrolled in UPBEAT, a psychogeriatric treatment program: a pilot study. *J Gerontol A Biol Sci Med Sci* **2004**, *59*, 1068-1075, doi:10.1093/gerona/59.10.m1068.
