# Supplementary material for: The microRNA-34a-Induced Senescence-Associated Secretory Phenotype (SASP) Favors Vascular Smooth Muscle Cells Calcification
Source: Int J Mol Sci. 2020 Jun 23;21(12):4454. doi: 10.3390/ijms21124454 (PMC7352675; doi:10.3390/ijms21124454)
Supplement: Supplementary file 1 [file ijms-21-04454-s001.zip › Supplementary Materials/Supplementary Table 1.docx]

**Table S1**. **The overexpression of miR-34a in HASMCs induces the expression of several SASP factors.**

| **Name of Protein fold induction P value**  **(miR-34a/CTRL)** |
| --- |
| \| ENA-78 \| 1.20 \| 0.3148 \| \| --- \| --- \| --- \| \| GCSF \| 1.30 \| 0.1039 \| \| GM-CSF \| 1.25 \| 0.1120 \| \| GRO-alfa \| 1.25 \| **0.0118** \| \| I-309 \| 1.24 \| ***0.0501*** \| \| IL-1alfa \| 1.23 \| 0.0884 \| \| IL-1beta \| 1.20 \| 0.2522 \| \| IL-5 \| 1.25 \| 0.0972 \| \| IL-6 \| 1.25 \| **0.0375** \| \| IL-7 \| 1.28 \| 0.0932 \| \| IL-8 \| 1.23 \| 0.1446 \| \| IL-10 \| 1.34 \| **0.0205** \| \| IL-12 \| 1.24 \| ***0.0502*** \| \| IL-13 \| 1.27 \| **0.0262** \| \| IL-15 \| 1.23 \| 0.7968 \| \| IFN-gamma \| 1.21 \| 0.1848 \| \| MCP-3 \| 1.51 \| 0.1532 \| \| MDC \| 1.20 \| 0.2379 \| \| MIG \| 1.24 \| 0.0861 \| \| MIP-1delta \| 1.29 \| **0.0140** \| \| RANTES \| 1.23 \| 0.1636 \| \| SCF \| 1.23 \| 0.1504 \| \| SDF-1 \| 1.20 \| 0.1372 \| \| Angiogenin \| 1.22 \| 0.3999 \| \| Oncostatin M \| 1.21 \| 0.3995 \| \| Thrombopoietin \| 1.24 \| 0.9865 \| \| PDGF-BB \| 1.21 \| 0.0872 \| \| BDNF \| 0.66 \| 0.2743 \| \| Eotaxin-3 \| 1.22 \| 0.1956 \| \| FGF-4 \| 1.27 \| 0.1714 \| \| FGF-6 \| 1.21 \| 0.2538 \| \| FGF-7 \| 1.21 \| 0.1247 \| \| Flt-3 Ligand \| 1.21 \| 0.1424 \| \| GCP-2 \| 1.42 \| 0.1185 \| \| HGF \| 1.32 \| 0.3033 \| \| IGFBP-3 \| 1.33 \| **0.0436** \| \| MCP-4 \| 1.30 \| 0.2229 \| \| MIF \| 1.21 \| 0.3334 \| \| MIP-3alfa \| 1.28 \| 0.1779 \| \| NT-3 \| 1.20 \| 0.2306 \| \| NT-4 \| 1.34 \| 0.1430 \| \| Osteopontin \| 1.23 \| 0.3343 \| \| PARC \| 1.31 \| **0.0270** \| \| PIGF \| 1.26 \| ***0.0525*** \| \| TIMP-1 \| 1.42 \| 0.1152 \| \| TIMP-2 \| 1.45 \| **0.0461** \| |

ENA-78=Epithelial neutrophil- activating protein 78; GCSF=Granulocyte-colony stimulating factor; GM-CSF=Granulocyte-Macrophage Colony-Stimulating Factor; GRO=growth-regulated oncogene; GRO-alfa=growth-regulated oncogene-alfa; I-309=human cytokine I-309; IL-1alfa=Interleukin 1 Alpha; IL-1beta= Interleukin 1 Beta; IL-5= Interleukin 5; IL-6=Interleukin 6; IL-7=Interleukin 7; IL-8=Interleukin 8; IL-10=Interleukin 12; IL-13=Interleukin 13; IL-15=Interleukin 15; IFN-gamma=Interferon gamma; MCP-3=Monocyte chemotactic protein 3; MDC=Macrophage-derived chemokine; MIG=monokine induced by gamma interferon; MIP-1delta=Macrophage Inflammatory Proteins-1 delta; Rantes=Regulated on Activation. Normal T Cell Expressed and Secreted; SCF= Stem-cell factor; SDF-1=stromal cell-derived factor 1; PDGF-BB=Human Platelet-Derived Growth Factor-BB; BDNF=Brain-derived neurotrophic factor; FGF-4=Fibroblast Growth Factor-4; FGF-6=Fibroblast growth factor-6; FGF-7= Fibroblast growth factor-7; Flt-3 Ligand= Fms-like tyrosine kinase 3 ligand; GCP-2=Graded Care Profile 2; HGF=Hepatocyte growth factor; IGFBP-3=Insulin-like Growth Factor Binding Protein 3; MCP-4=Monocyte Chemoattractant Protein 4; MIF=Macrophage migration inhibitory factor; MIP-3alfa=Macrophage inflammatory protein-3 alpha; NT-3=Neurotrophin-3; NT-4=Neurotrophin-4; PARC=Pulmonary and activation-regulated chemokine; PIGF=Placental growth factor; TIMP-1=Metallopeptidase Inhibitor 1; TIMP-2=Metallopeptidase Inhibitor 2.
