# Supplementary material for: The microRNA-34a-Induced Senescence-Associated Secretory Phenotype (SASP) Favors Vascular Smooth Muscle Cells Calcification
Source: Int J Mol Sci. 2020 Jun 23;21(12):4454. doi: 10.3390/ijms21124454 (PMC7352675; doi:10.3390/ijms21124454)
Supplement: Supplementary file 1 [file ijms-21-04454-s001.zip › Supplementary Materials/Supplementary Table 2_.docx]

**Table S2. Biochemical, metabolic and anthropometric** **variables of healthy subjects divided in three age groups.**

| **Variables ≤45 yrs 46-64 yrs ≥65 yrs P trend**  **n 31 54 43** |
| --- |
| \| **Sex (n/% male)** \| 16/51.61 \| 28/51.85 \| 17/39.53 \| **0.0437** \| \| --- \| --- \| --- \| --- \| --- \| \| **Height (cm)** \| 171.84±8.43 \| 167.56±7.58 \| 161.33±8.35 \| **<0.0001** \| \| **Weight (kg)** \| 75.31±15.72 \| 77.65±12.87 \| 69.97±10.11 \| 0.1200 \| \| **BMI (Kg/m^2^)** \| 25.37±4.29 \| 27.70±4.52 \| 26.83±3.06 \| 0.1700 \| \| **WC (cm)** \| 85.06±13.3 \| 92.57±11.97 \| 94.33±8.75 \| **0.0020** \| \| **H (cm)** \| 99.73±10.29 \| 104.43±9.23 \| 103.00±7.93 \| 0.1800 \| \| **W/H** \| 0.85±0.090 \| 0.89±0.08 \| 0.92±0.07 \| **0.0030** \| \| **RBC (10^6^/uL)** \| 4.91±0.45 \| 4.63±0.39 \| 4.71±0.38 \| 0.0600 \| \| **WBC (10^3^/uL)** \| 6.88±1.610 \| 6.49±1.78 \| 5.86±1.38 \| **0.0160** \| \| **Monocytes** \| 6.30(5.20;7.50) \| 6.20(5.70;6.90) \| 6.50(5.50;7.30) \| 0.5900 \| \| **Triglycerides (mg/dL)** \| 71.50(50;118.00) \| 84.50(55.00;109.00) \| 123.50(87;180) \| **0.0010** \| \| **Cholesterol (mg/dL)** \| 199.06±34.00 \| 218.83±42.29 \| 226.27±43.61 \| **0.0100** \| \| **HDL (mg/dL)** \| 53.29±12.89 \| 59.37±14.91 \| 58.07±16.52 \| 0.2100 \| \| **LDL (mg/dL)** \| 117.34±28.52 \| 129.41±40.02 \| 134.99±36.66 \| 0.0600 \| \| **ApoAI (mg/dL)** \| 166.77±28.02 \| 178.46±33.58 \| 184.40±31.56 \| **0.0320** \| \| **ApoB (mg/dL)** \| 101.67±36.96 \| 105.61±30.88 \| 114.33±39.48 \| 0.1600 \| \| **Insulin (uiU/mL)** \| 4.78(3.8;6.59) \| 4.21(2.95;5.71) \| 5.26(3.6;7.15) \| 0.6100 \| \| **Glucose (mg/dL)** \| 89.90±8.53 \| 96.81±10.67 \| 96.50±8.74 \| **0.0090** \| \| **HOMA Index** \| 1.05(0.87;1.50) \| 0.99(0.72;1.51) \| 1.22(0.78;10.63) \| 0.9800 \| \| **HGB (g/dL)** \| 14.24±1.17 \| 14.27±1.26 \| 14.04±1.09 \| 0.5200 \| \| **HbA1c (%)** \| 5.46±0.37 \| 5.76±0.47 \| 5.89±0.45 \| **0.0002** \| \| **GGT (U/L)** \| 44.4±15.41 \| 46.13±18.22 \| 50.60±15.64 \| 0.1600 \| \| **AST (U/L)** \| 19.00(16;24) \| 19.00(15;24) \| 21.00(19;27) \| **0.0240** \| \| **ALT (U/L)** \| 36.00(30;41) \| 36.00(32;41) \| 36.00(31;42) \| 0.9300 \| \| **FLI** \| 47.09±27.32 \| 58.04±26.67 \| 66.06±19.11 \| **0.0040** \| \| **Transferrin (mg/dL)** \| 284.87±39.93 \| 246.22±41.07 \| 242.23±45.61 \| **0.0002** \| \| **Ferritin (ng/mL)** \| 43.95(16.30;115.30) \| 100.75(52.40;162) \| 73.85(53.50;183.70) \| **0.0029** \| \| **Azotemia (mg/dL)** \| 31.00(27;37) \| 38.00(33;43) \| 39.50(35;49) \| **<0.0001** \| \| **Creatinine (mg/dL)** \| 0.83±0.20 \| 0.81±0.19 \| 0.92±0.20 \| 0.0800 \| \| **Fibrinogen** \| 241.00(202;282) \| 294.00(250.5;326) \| 290.00(261;341) \| **0.0016** \| \| **Bilirubin** \| 0.60(0.40;0.80) \| 0.70(0.50;0.90) \| 0.70(0.60;0.90) \| 0.1200 \| \| **PAI-1 (ng/mL)** \| 17.88(12.28;25.39) \| 17.35(13.19;27.20) \| 19.77(11.9;30.05) \| 0.8700 \| \| **CRP (mg/L)** \| 1.20(0.60;2.01) \| 1.60(0.67;3.37) \| 3.18(1.79;6.48) \| **0.0001** \| \| **IL6 (pg/mL)** \| 1.09(0.82;1.49) \| 1.37(0.97;2.6) \| 2.42(2.02;3.55) \| **<0.0001** \| \| **IL 8 (pg/ml)** \| 10.82(8.54;13.73) \| 15.43(10.77;20.11) \| 15.25(11.62;18.20) \| **0.0014** \| \| **miR-34a** \| 4.58(1.85;8.73) \| 5.05(2.05;2.27) \| 6.4(2.62;11.1) \| 0.2721 \| \| **Ipertension (n/%)** \| 3/9.70 \| 12/22.20 \| 17/56.70 \| **<0.0001** \| \| **Smoking (n/%)** \| 9/29.00 \| 14/25.90 \| 4/13.30 \| 0.1500 \| |

Variables with Gaussian distribution are expressed as mean ± SD; variables with skewed distribution as median and interquartile range. In bold statistically significant P trend value. BMI=body mass index; WC=Waist Circumference; H=Hip; W/H=Waist/Hip ratio; WBC=White blood cells. RBC=Red blood cells; HDL=High Density Lipoprotein; LDL=Low Density Lipoprotein; ApoAI=Apolipoprotein AI; ApoB=Apolipoprotein B; HGB=Hemoglobin; HbA1c=glycated hemoglobin; GGT=Gamma Glutamyl transferase; AST=Aspartate transaminase; ALT=Alanine transferase; PAI*‐*1=Plasminogen activator inhibitor‐1; CRP= C-reactive Protein; IL6=Interleukin 6; IL8=Interleukin 8; FLI=Fatty Liver index;
