# Supplementary material for: The microRNA-34a-Induced Senescence-Associated Secretory Phenotype (SASP) Favors Vascular Smooth Muscle Cells Calcification
Source: Int J Mol Sci. 2020 Jun 23;21(12):4454. doi: 10.3390/ijms21124454 (PMC7352675; doi:10.3390/ijms21124454)
Supplement: Supplementary file 1 [file ijms-21-04454-s001.zip › Supplementary Materials/Supplementary Table 3.docx]

**New Table S3**. **List of primers used for quantitative RT-PCR.**

| **Oligo name Nucleotide Sequence (5’→3’)** |
| --- |
| \| Human_IL6_forward \| ACAAAAGTCCTGATCCAGTTCC \| \| --- \| --- \| \| Human_IL6_reverse \| GACTGCAGGAACTCCTTAAAGC \| \| Human_GAPDH_forward \| AATCCCATCACCATCTTCCAG \| \| Human_GAPDH_reverse \| AAATGAGCCCCAGCCTTC \| \| Human_p16_forward \| CTTCCTGGACACGCTGGT \| \| Human_p16_reverse \| CAAACCCACAAATGGTTTCC \| \| Human_p21_forward \| TGTCACTGTCTTGTACCCTTG \| \| Human_p21_reverse \| GGCGTTTGGAGTGGTAGAA \| \| Human_HPRT_forward \| CCTGGCGTCGTGATTAGTGA \| \| Human_HPRT_reverse \| TGATGAAGGAGATGGGAGGC \| \| Mouse_ IL6_forward \| GCTACCAAACTGGATATAATCAGGA \| \| Mouse_IL6_reverse \| CCAGGTAGCTATGGTACTCCAGAA \| \| Mouse_Hprt_forward \| GGAGCGGTAGCACCTCCT \| \| Mouse_Hprt_reverse \| CCAAATCCTCGGCATAATGA \| \|  \|  \| |
