# Supplementary figures and images for: The microRNA-34a-Induced Senescence-Associated Secretory Phenotype (SASP) Favors Vascular Smooth Muscle Cells Calcification
Source: Int J Mol Sci. 2020 Jun 23;21(12):4454. doi: 10.3390/ijms21124454 (PMC7352675; doi:10.3390/ijms21124454)

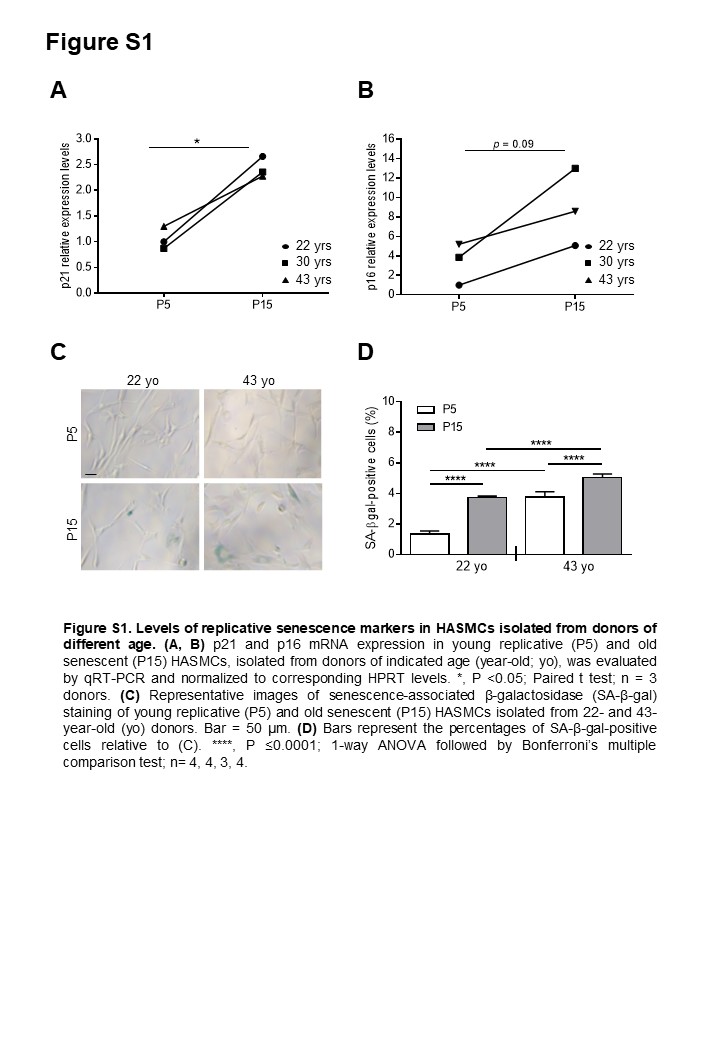

Supplement: Supplementary file 1 [file ijms-21-04454-s001.zip › Supplementary Materials/Figure S1.jpg]

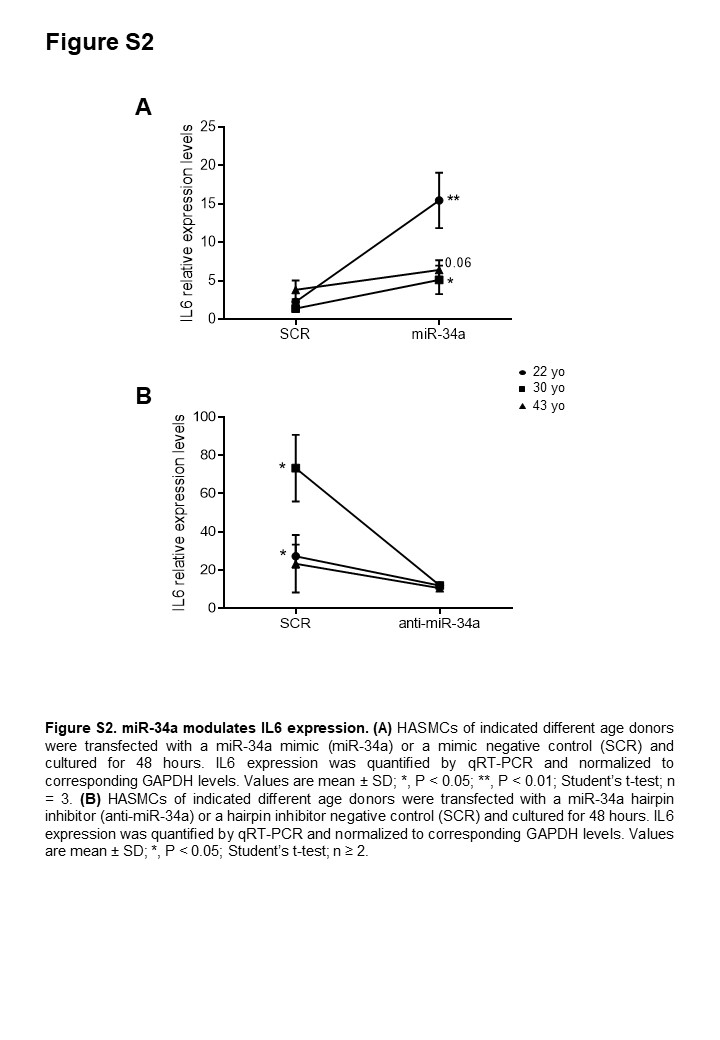

Supplement: Supplementary file 1 [file ijms-21-04454-s001.zip › Supplementary Materials/Figure S2.jpg]

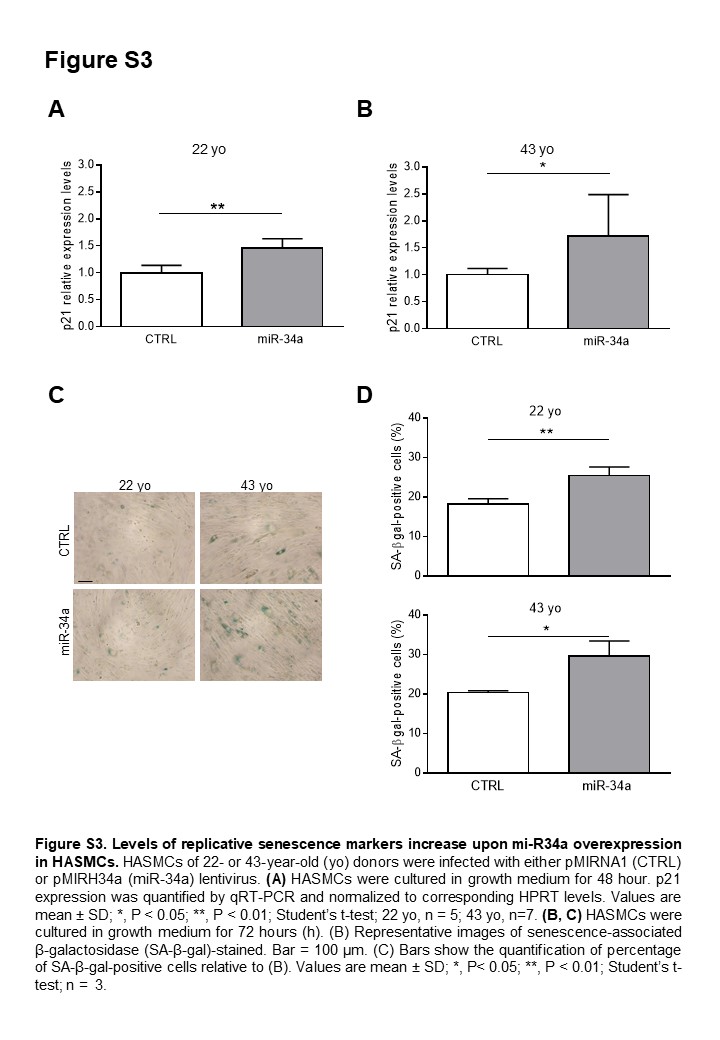

Supplement: Supplementary file 1 [file ijms-21-04454-s001.zip › Supplementary Materials/Figure S3.jpg]

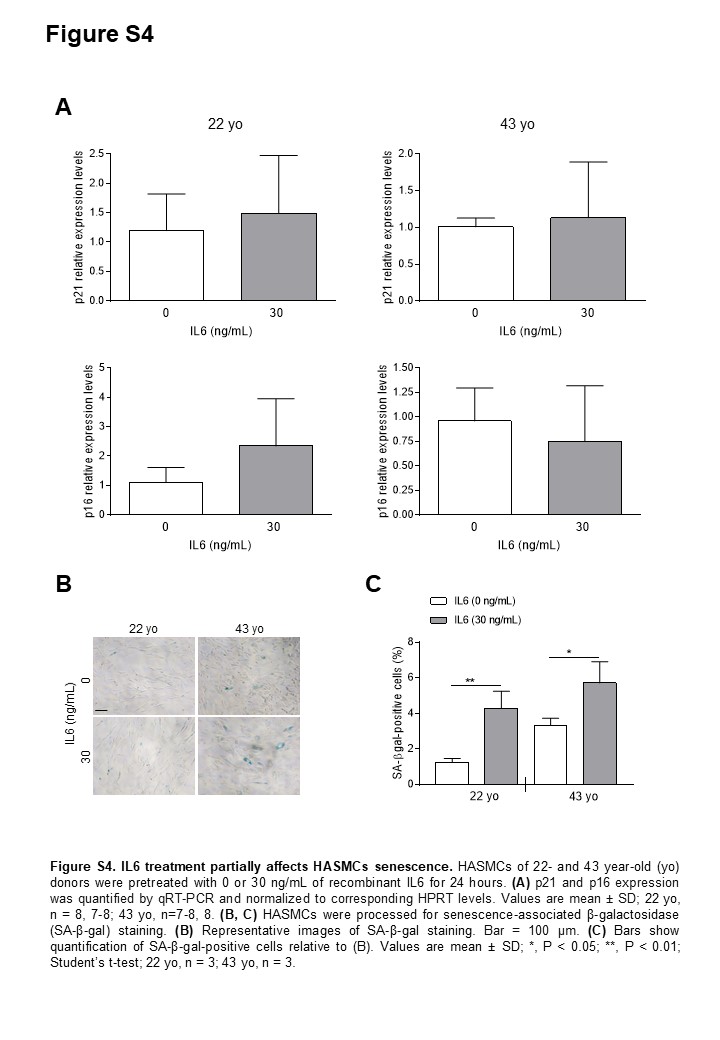

Supplement: Supplementary file 1 [file ijms-21-04454-s001.zip › Supplementary Materials/Figure S4.jpg]

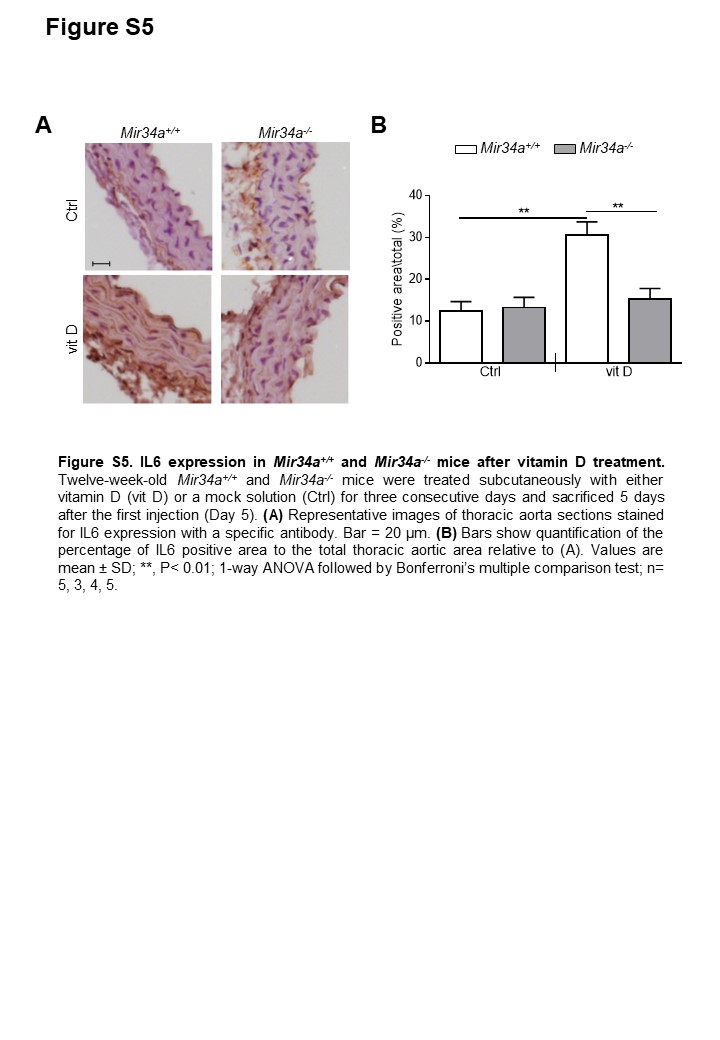

Supplement: Supplementary file 1 [file ijms-21-04454-s001.zip › Supplementary Materials/Figure S5.jpg]
